# HyperSight^TM^-CBCT based monitoring of sarcopenia during definitive radiotherapy of prostate cancer: a longitudinal feasibility study

**DOI:** 10.1186/s13014-026-02856-3

**Published:** 2026-05-29

**Authors:** Victor Siefert, Joana V. Bettenhausen, Alicia S. Bicu, Ralf Schmidt, Marvin Willam, Miriam Eckl, Florian Stieler, Michael Ehmann, Daniel Buergy, Jens Fleckenstein, Bettina Beuthien-Baumann, Heinz-Peter Schlemmer, Matthias F. Froelich, Stefan O. Schoenberg, Frank A. Giordano, Judit Boda-Heggemann, Constantin Dreher

**Affiliations:** 1 Department of Radiation Oncology, University Medical Centre Mannheim, Medical Faculty Mannheim, University of Heidelberg, Theodor-Kutzer Ufer 1-3, 68167 Mannheim, Germany; 2 DKFZ-Hector Cancer Institute, University Medical Centre Mannheim, Mannheim, Germany; 3 Mannheim Institute for Intelligent Systems in Medicine (MIiSM), University of Heidelberg, Mannheim, Germany; 4 Junior Research Group “Intelligent Imaging for Adaptive Radiotherapy”, MIiSM, University of Heidelberg, Mannheim, Germany; 5 Junior Research Group “Image and Surface Guided Radiotherapy”, MIiSM, University of Heidelberg, Mannheim, Germany; 6 Department of Radiology, German Cancer Research Center (DKFZ), Heidelberg, Germany; 7 Department of Radiology and Nuclear Medicine, University Medical Centre Mannheim, Medical Faculty Mannheim, University of Heidelberg, Mannheim, Germany

## Abstract

**Background:**

Sarcopenia and treatment-related muscle loss are associated with increased toxicity and reduced resilience in cancer patients. HyperSight™ cone-beam CT (CBCT) provides improved soft-tissue contrast compared with conventional CBCT and may enable reliable longitudinal skeletal-muscle monitoring during image-guided radiotherapy (IGRT). This study evaluated the feasibility of HyperSight™ CBCT-based muscle assessment in prostate cancer patients and explored the clinical relevance of intratreatment muscle decline.

**Methods:**

Eighty-eight patients with non-metastatic prostate cancer were treated on an Ethos^®^ system with HyperSight™ CBCT. Patients received ultrahypofractionated (UHF) or normo-/moderately hypofractionated (NF/MHF) radiotherapy; 28% received androgen-deprivation therapy. Cross-sectional muscle area (CSA) and radiodensity (HU) were assessed on planning CT (PCT) and serial CBCTs (first, second, last fraction [FF, SF, LF]); HU values were z-standardized to subcutaneous fat (zHU). Agreement between PCT and CBCT was evaluated using concordance correlation coefficients (CCC), regression, and Bland-Altman analysis. Longitudinal changes were analyzed using mixed-effects models, and associations with acute genitourinary/gastrointestinal (GU/GI) toxicity (RTOG ≥ 2) using regression models.

**Results:**

HyperSight™ CBCT showed excellent agreement with PCT for CSA (CCC = 0.981). The NF/MHF cohort showed a significant longitudinal CSA decline (FF-LF: -153.0 mm², *p* = 0.021), whereas no significant within-cohort change was observed in UHF. Muscle density (zHU) decreased significantly in both cohorts (NF/MHF: *p* < 0.001; UHF: *p* = 0.0017). Weight loss and age were independently associated with zHU change, while no independent predictors of CSA decline were identified. In the NF/MHF cohort, CSA loss > 5% was associated with acute GU toxicity in univariate analysis (OR 4.90, *p* = 0.033; Fisher’s exact *p* = 0.014), but not after adjustment for age, weight loss, ECOG performance status, and ADT; no associations were found for GI toxicity or in the UHF cohort.

**Conclusions:**

HyperSight™ CBCT enables quantitative skeletal-muscle assessment during prostate radiotherapy and detects intratreatment reductions in muscle density, which may represent an early imaging marker of muscle decline. Muscle loss showed an exploratory association with acute GU toxicity in NF/MHF patients in univariate analysis, but not after adjustment. These findings support the feasibility of CBCT-based muscle monitoring and warrant further evaluation in larger cohorts.

**Clinical trial number:**

Not applicable.

## Introduction

Computed tomography (CT)-based assessment of skeletal muscle composition has emerged as a reliable method for diagnosing sarcopenia, a condition characterized by progressive loss of muscle mass and function [1, 2]. In oncologic populations, particularly among patients receiving radiotherapy, reduced muscle quantity or quality has been linked to unfavorable outcomes, including higher treatment toxicity, weight loss, and decreased overall survival [3, 4]. While various approaches - such as dual-energy X-ray absorptiometry (DXA), bioimpedance analysis, and MRI - are used to evaluate muscle health, quantitative CT analysis of cross-sectional muscle area (CSA) and radiodensity has become a rising adopted tool in oncologic imaging research [5, 6].

However, most CT-based evaluations are limited to baseline measurements, focusing on the identification of pre-existing muscle depletion at the start of therapy [7–9]. Implementing a longitudinal imaging strategy could provide valuable insights into treatment-related alterations in body composition. This is particularly relevant in curative radiotherapy for prostate cancer, where muscle status has been identified as an independent prognostic factor for overall survival [10–12].

Routine image-guided radiotherapy (IGRT) offers an opportunity to achieve such longitudinal monitoring through cone-beam computed tomography (CBCT) imaging. These daily imaging datasets can provide serial information on soft-tissue changes without additional radiation exposure, thereby supporting a more patient-centered and adaptive approach to treatment follow-up. Moreover, CBCT-based body composition analysis may help to identify therapy-induced changes influenced by fractionation schedules or concurrent androgen deprivation therapy (ADT), which itself has been associated with accelerated muscle loss and increased non-cancer mortality in prostate cancer [13, 14].

Previous feasibility studies have primarily focused on head and neck cancer cohorts, where CBCT has been used to quantify skeletal muscle changes during treatment [15]. However, standard CBCT suffers from limited soft-tissue contrast and significantly decreased image quality, reducing its precision for detailed body composition assessment, particularly in pelvic imaging, where tissue differentiation is inherently lower [16]. Additionally, prostate CBCT scans typically exclude the psoas muscle at the lumbar L3-L5 level, the conventional reference for sarcopenia evaluation [17]. As an alternative, pelvic and proximal thigh muscles have been reported to correlate with established lumbar muscle indices and may serve as suitable surrogate regions for longitudinal pelvic muscle monitoring [18].

Recently, technological advancements have begun to overcome these limitations. The HyperSight™ CBCT system, integrated into the Ethos^®^ linear accelerator (Varian, Siemens Healthineers), offers promising improvements in image quality and technical performance [19–21]. Notably, Hounsfield unit (HU) values obtained from HyperSight™ CBCT have been shown to be comparable to those from diagnostic fan-beam CT [22, 23]. These innovations open the possibility for more reliable, quantitative assessments of muscle mass and density directly from fractional CBCT scans, expanding the diagnostic role of routine IGRT beyond anatomical alignment.

In light of these advancements, the diagnostic potential of high-quality CBCT imaging within precision radiotherapy continues to grow. This study therefore aims to evaluate the feasibility and reliability of CBCT-derived muscle measurements in prostate cancer patients and to explore their clinical relevance by assessing and correlating longitudinal changes in muscle mass and density with treatment-related outcomes such as weight loss and acute toxicity at las.

### Patients and study design

This prospective registry study was approved by the Ethics Committee of the Medical Faculty Mannheim, University of Heidelberg, Germany. It initially included 96 patients with histologically confirmed, localized to locally advanced prostate carcinoma without lymph node or distant metastases who underwent definitive image-guided radiotherapy from 02/2024 to 04/2025. All patients provided written informed consent prior to study inclusion. Depending on the treatment regimen, patients received either normofractionated/mild hypofractionated radiotherapy (30 fractions, NF), moderately hypofractionated radiotherapy (20 fractions, MHF), or ultrahypofractionated radiotherapy (5 fractions, UHF). ADT was administered either before or during radiotherapy in about a quarter of the cohort according to institutional guidelines and individual tumor risk profiles (more frequently in the NF/MHF group).

All patients were treated using the Ethos^®^ linear accelerator system (Varian Medical Systems, Siemens Healthineers), which employs HyperSight™ CBCT imaging for daily image guidance. After excluding six patients who were not eligible because treatment intent had been misclassified (postoperative prostate bed irradiation, *n* = 3) or because they presented with nodal metastasis (*n* = 1) or oligometastatic disease (*n* = 2), a total of 88 patients were eligible for final analysis (evenly distributed between the UHF and the NF/MHF treatment groups). No elective pelvic lymph node irradiation was performed in this cohort.

Clinical and demographic data were extracted from the electronic health records. Detailed baseline characteristics are summarized in Table 1.

**Table 1:** Baseline characteristics

Characteristics	All (*n* = 88)	UHF (*n* = 44)	NF/MHF (*n* = 44)
Age (years), mean ± SD	72.8 ± 8.1	71.1 ± 7.8	74.5 ± 8.1
Height (cm), mean ± SD	178.2 ± 6.7	178.5 ± 6.2	177.9 ± 7.1
Weight (kg), mean ± SD	85.8 ± 13.7	85.5 ± 11.4	86.0 ± 15.8
T stage, n (%)			
T1	61 (69.3)	33 (75.0)	28 (63.6)
T2	17 (19.3)	8 (18.2)	9 (20.5)
T3	10 (11.4)	3 (6.8)	7 (15.9)
ADT, n (%)	25 (28.4)	6 (13.6)	19 (43.2)
RT concepts			
5 Fractions, n (%)	44 (50.0)	44 (100)	-
20 Fractions, n (%)	31 (35.2)	-	31 (70.5)
30 Fractions, n (%)	13 (14.8)	-	13 (29.5)
Acute Toxicity Events			
GU ≥ 2	15 (17.0)	2 (4.5)	13 (29.5)
GI ≥ 2	7 (8.0)	0 (0.0)	7 (15.9)

Comparison of demographic and clinical baseline characteristics between patients treated with ultrahypofractionated radiotherapy (UHF) and normofractionated&mild/moderately hypofractionated radiotherapy (NF/MHF). Continuous variables are presented as mean ± standard deviation (SD), and categorical variables as number (percentage). Acute toxicity events are reported as the number and proportion of patients with acute genitourinary (GU) and gastrointestinal (GI) toxicity ≥ grade 2 according to RTOG criteria. Abbreviations: UHF, ultrahypofractionation; NF/MHF, normo-/moderate hypofractionation; SD, standard deviation; ADT, androgen deprivation therapy; GU, genitourinary; GI, gastrointestinal

#### CT and CBCT acquisition protocols

HyperSight™ CBCT imaging was performed on an Ethos^®^ linear accelerator (Varian, Siemens Healthineers). Image acquisition used the standard Pelvis protocol with a tube voltage of 120 kVp, a tube current-exposure product of 468 mAs, a slice thickness of 2 mm, a pixel size of 1.0507 × 1.0507 mm, and a CTDIvol of 8.89 mGy. Images were reconstructed using one of two available approaches: either the standard iterative CBCT algorithm (iCBCT) or the iCBCT Acuros reconstruction mode. While iCBCT reduces noise through iterative statistical reconstruction, iCBCT Acuros adds advanced scatter correction and enhanced modeling, providing superior HU accuracy and image uniformity at the cost of longer reconstruction times [24–26].

Planning CT (PCT) scans were acquired on a Brilliance BigBore system (Philips). Acquisition parameters included a tube voltage of 120 kVp, a mean tube current-time product of 232.97 mAs, slice thickness of 2 mm for patients in the ultrahypofractionated group and 3 mm for those in the normo- or moderately hypofractionated group, and an in-plane pixel size of 1.17 × 1.17 mm. The mean CTDIvol across all planning CTs was 13.87 mGy.

#### Image segmentation

HyperSight™ CBCT datasets were imported into the analysis software as Digital Imaging and Communications in Medicine (DICOM) files. The baseline planning CT (PCT) and fractional CBCTs acquired at predefined treatment timepoints (first (FF), second (SF), and last fraction (LF)) were investigated as follows:

Part A: Regional assessment to evaluate body composition.

In order to assess the skeletal muscle area in the prostate cancer radiotherapy treatment region, the association between skeletal muscle area in the pelvic region and the gold standard for body composition analysis, skeletal muscle area at the lumbar level, was first investigated for the complete population. Prior studies have demonstrated strong correlations between skeletal muscle area measured at L3 and adjacent lumbar levels, including L4 [27], and also with pelvic and proximal thigh muscles [18]. As PCT scans are performed with an extended field of view up to L4 cranially, compared to S1 for CBCT scans, a translational body composition correlation analysis was performed at the PCT scan between L4 and the selected pelvic surrogate muscle groups (see description in Part B). For this purpose, the summed cross-sectional area (CSA) of all segmented pelvic and proximal thigh muscles on planning CT was correlated with total skeletal muscle CSA at the L4 level, correspondingly adapted of L3 by Zhao et al. [28].

Part B: Clinical analysis of body composition in the treatment region.

**Fig. 1:**
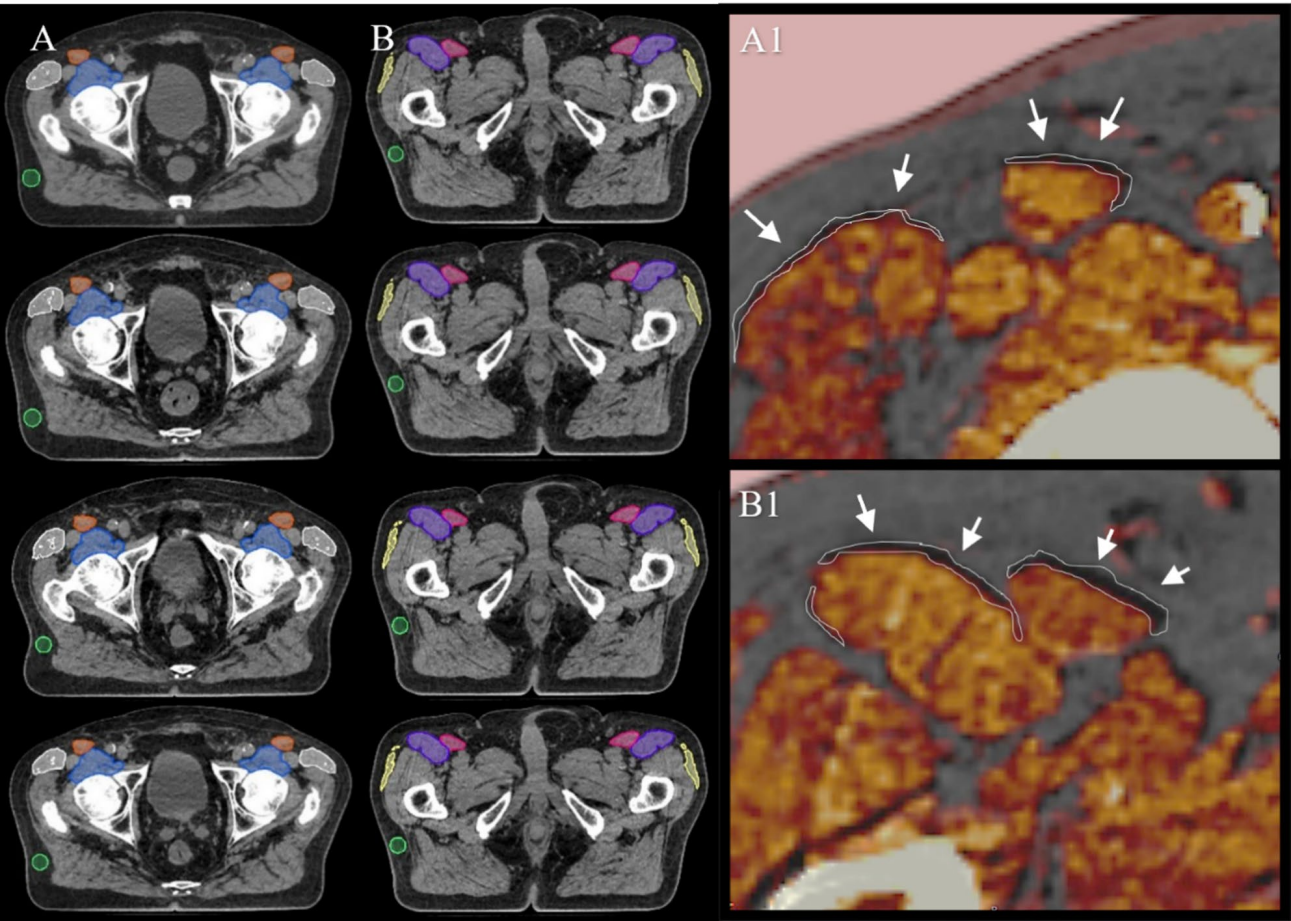
Axial slices illustrating proximal (**A**) and distal (**B**) pelvic muscle segmentations and longitudinal changes in muscle morphology during radiotherapy. Column A shows the planning CT at the top, followed by the 1st, 2nd, and 20th cone-beam CTs (CBCTs) for a patient with moderately hypofractionated radiotherapy; column B displays the corresponding distal levels. Proximal muscles include the tensor fasciae latae, sartorius, and iliopsoas, while distal muscles comprise the tensor fasciae latae, sartorius, and rectus femoris. A gluteal subcutaneous fat ROI (green) was additionally delineated for Hounsfield Unit (HU) normalization and stability assessment. The overlay panels A1 and B1 depict magnified views of the 1st and 20th CBCTs, with white contours outlining muscle regions from the initial scan that have decreased over time. All imaging was performed using a Philips Brilliance BigBore CT scanner for planning and HyperSight™ CBCT on the Varian Ethos^®^ system for treatment imaging

The skeletal muscle area in the prostate cancer radiotherapy treatment region was investigated at two levels. At each of the two assessed pelvic levels, three representative pelvic muscles per side were segmented as follows: the tensor fasciae latae, sartorius, and iliopsoas at the proximal (femoral head) level, and the tensor fasciae latae, sartorius, and rectus femoris at the distal (lesser trochanter) level. In addition, a subcutaneous gluteal fat ROI was contoured to assess HU stability and normalization across timepoints. Representative axial images illustrating proximal and distal muscle segmentations and longitudinal changes in muscle morphology during radiotherapy are shown in Fig. 1.

Segmentation Methodology:

Regions of interest (ROIs) were first semi-automatically segmented using 3D Slicer (Version 5.8.1, https://www.slicer.org). A HU threshold range of − 29 to + 150 HU was applied to isolate skeletal muscle tissue [29]. Secondly, the segmentations were manually refined, if necessary, and/or approved by two observers in consensus (R1 with 9 years and R2 with 4 years of experience in CT imaging) for the complete cohort. To additionally assess the general reliability of finalized CBCT segmentations, intraobserver reliability (in a consensus repeat) was evaluated in 20% of the investigated population (a subset of *n* = 18).

Cross-sectional muscle area (CSA, mm²) and mean tissue density (HU) were automatically extracted from the axial images.

HU values were standardized using a z-score transformation relative to a reference region of interest (ROI) in subcutaneous fat, in analogy to conventional z-score normalization approaches [30]. The z-score for each muscle ROI was then calculated as

$$\:\mathrm{z}\:=\:(\mathrm{H}\mathrm{U}\_\mathrm{m}\mathrm{u}\mathrm{s}\mathrm{c}\mathrm{l}\mathrm{e}\:-\:\mathrm{m}\mathrm{e}\mathrm{a}\mathrm{n}\_\mathrm{f}\mathrm{a}\mathrm{t})\:/\:\mathrm{S}\mathrm{D}\_\mathrm{f}\mathrm{a}\mathrm{t}$$

Because PCT and CBCT differed in acquisition and reconstruction parameters, longitudinal analyses for both CSA and zHU were limited to CBCT timepoints (FF, SF, and LF). Changes were assessed relative to FF, and direct statistical comparisons between PCT and CBCT were not performed.

### Statistical analysis

All data analysis was conducted in R (version 4.4.2) as follows.

Pearson correlation coefficient analysis and linear regression analysis were performed to assess the association of different CSA levels at L4 and in the pelvic treatment region for PCT imaging. 

Intraobserver reliability of muscle CSA measurements in CBCT imaging was assessed using the intraclass correlation coefficient (ICC; two-way random-effects model, absolute agreement, single measurements; ICC (2,1)).

To assess agreement of CSA between CT (PCT) and CBCT (FF), mean CSA values from both sides (right/left) were calculated for six muscle groups. Agreement was quantified using the Pearson correlation and the concordance correlation coefficient (CCC). Bland-Altman plots were used to visualize systematic differences. Linear regression analysis was performed between PCT and FF, using the mean CSA averaged across all segmented muscles. All patients were analyzed within a single model without stratification by treatment regimen. The resulting regression equation was subsequently applied to later CBCT timepoints (e.g., SF, LF, or intermediate fractions such as the 5th or 20th scan), depending on the respective treatment schedule.

Changes in skeletal muscle CSA and muscle density (HU) were analyzed using linear mixed-effects models with time point as fixed effect and patient as random effect. Analyses were performed on change-from-baseline values with the first treatment fraction (FF) serving as baseline (ΔCSA and ΔzHU vs. FF). Changes were defined as Δ = FF - timepoint, so that positive values represent a decrease from baseline. A random intercept was included to account for inter-individual differences in baseline measurements. Models were estimated via restricted maximum likelihood. Pairwise post-hoc comparisons between time points were adjusted for multiple testing using the Tukey method. Statistical significance was defined as *p* < 0.05 (two-sided).

To further explore determinants of intratreatment muscle change, additional univariate and multivariate linear regression analyses were performed using ΔCSA (%) and ΔzHU between the first and last treatment fraction as dependent variables. Candidate predictors included weight loss during radiotherapy, age, ECOG performance status, ADT, fractionation regimen (NF/MHF vs. UHF), and the occurrence of acute genitourinary (GU) or gastrointestinal (GI) toxicity of grade ≥ 2 until last fraction of radiotherapy. Multivariable models included all covariates simultaneously. Results are reported as regression coefficients (β) with 95% confidence intervals and corresponding p-values.

To explore potential predictors of acute GU and GI toxicity during the radiotherapy course, patients were dichotomized according to the occurrence of RTOG grade ≥ 2 events. Associations with CSA decline > 5% between baseline planning CT and the last fraction were first examined separately for GU and GI endpoints using Fisher’s exact test, given the limited sample size and small cell counts. The 5% threshold for CSA loss was chosen as it has been proposed in previous oncologic studies to represent a clinically meaningful decline, while minimizing the influence of measurement variability. In addition, univariate logistic regression models were fitted with GU or GI toxicity (RTOG ≥ 2) as the dependent variable and clinical covariates (CSA loss > 5%, CSA loss [%], weight loss during radiotherapy, patient age, ECOG performance status, and use of ADT) as independent variables. Separate multivariate logistic regression models were fitted for dichotomous CSA loss (> 5%) and continuous CSA loss (%), each adjusted for weight loss during radiotherapy, patient age, ECOG performance status, and use of ADT. Results are reported as odds ratios (OR) with 95% confidence intervals and corresponding p-values.

## Results

### Part A: Regional assessment to evaluate body composition

The summed CSA of the analyzed pelvic muscles showed a significant correlation with total skeletal muscle CSA at L4 at the PCT (Pearson’s *r* = 0.713, 95% CI 0.592–0.803, *p* < 0.001). Linear regression confirmed this association (β = 1.32, R² = 0.509, *p* < 0.001), supporting the anatomical relevance of the selected pelvic surrogate muscle set.

### Part B: Clinical analysis of body composition in the treatment region

#### Reliability of pelvic surrogate muscle segmentation

Intraobserver reliability was very high for almost all measurements, with ICC values ranging from 0.885 to 0.999 (median 0.983). One distal sartorius measurement showed slightly lower agreement (ICC = 0.885), corresponding to good reliability.

#### Agreement between PCT and CBCT (FF) measurements

**Fig. 2:**
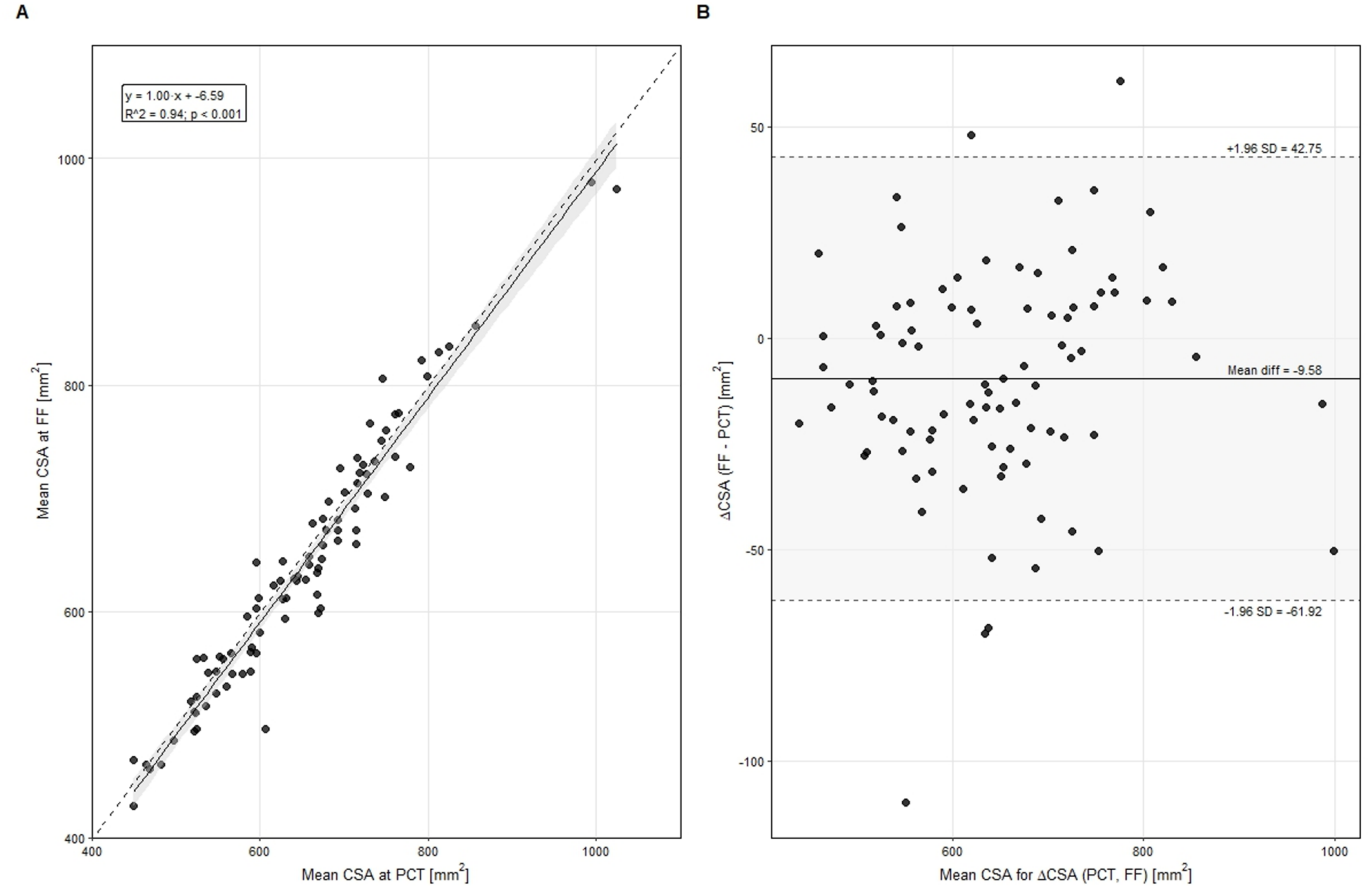
Agreement between planning CT (PCT) and first-fraction CBCT (FF) muscle CSA measurements. (**A**) Linear regression of mean CSA values from PCT and FF, including regression line and 95% confidence interval. (**B**) Bland-Altman plot showing the mean difference and limits of agreement between PCT and FF CSA measurements

Muscle CSA values obtained from the first CBCT (FF) showed excellent agreement with those from the planning CT (PCT) (CCC = 0.981, 95% CI 0.978–0.984). The Bland-Altman analysis demonstrated a minimal mean bias of 9.6 mm² with narrow limits of agreement (-100.7 to 119.9 mm²), indicating a high level of consistency without systematic deviation. Linear regression confirmed a strong correlation between PCT and FF measurements (R² = 0.94, *p* < 0.001; Fig. 2). To further test the robustness of this relationship, the regression model was applied to subsequent CBCT fractions, showing similarly high correlations (β = 0.92, R² = 0.92 for SF; β = 0.94, R² = 0.91 for LF).

#### Trajectory of cross-sectional muscle area and muscle density

**Fig. 3:**
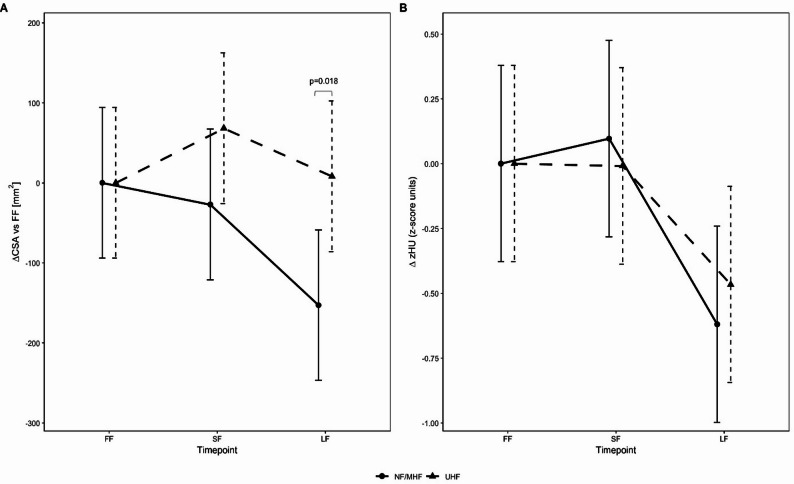
Trajectory of ΔCSA (change from baseline) during treatment. Estimated mean ΔCSA (± 95% CI) derived from a linear mixed-effects model, showing treatment-related changes in total muscle cross-sectional area relative to baseline. Separate lines indicate the ultrahypofractionated (UHF) and normo-/moderately hypofractionated (NF/MHF) cohorts. Abbreviations: ΔCSA, change from baseline in cross-sectional area; CSA, cross-sectional area; FF, first fraction; SF, second fraction; LF, last fraction; CBCT, cone-beam computed tomography; UHF, ultrahypofractionated; NF/MHF, normofractionated&mild-/moderately hypofractionated

Changes in muscle composition in CBCTs over the course of radiotherapy are displayed in Fig. 3. For total CSA, the ΔCSA at the last fraction (LF vs. FF) differed significantly between the two fractionation cohorts (UHF vs. NF/MHF; post-hoc *p* = 0.018). In the NF/MHF cohort, CSA decreased significantly from FF to LF (post-hoc Tukey-adjusted *p* = 0.021), whereas the corresponding within-cohort contrasts in the UHF cohort (SF-FF and LF-FF) were not significant.

For z-standardized HU, the ΔzHU values (vs. FF) did not differ significantly between the two cohorts over time (*p* = 0.40). In post-hoc Tukey-adjusted comparisons, both cohorts showed a significant decrease from FF to LF (UHF: *p* = 0.0017; NF/MHF: *p* < 0.0001), whereas the FF-SF contrast was not significant in either cohort. Table 2 lists the estimated mean differences (Δ) together with the post-hoc adjusted p-values for all predefined time-point contrasts.

**Table 2:** Changes in muscle CSA (A) and zHU (B) across predefined timepoints

(A) ΔCSA (mm²)				
Cohort	Contrast	ΔCSA (mm²)	95% CI	*p* (adj.)
NF/MHF	FF-SF	27.3	-106.9 to 161.4	0.8807
	FF-LF	153.0	18.8 to 287.2	0.0210*
	SF-LF	125.7	-8.4 to 259.9	0.0713
UHF	FF-SF	-68.0	-202.1 to 66.2	0.4560
	FF-LF	-8.1	-142.3 to 126.0	0.9887
	SF-LF	59.8	-74.3 to 194.0	0.5435

(B) ΔHU (z-score)				
Cohort	Contrast	ΔHU (z-score)	95% CI	*p* (adj.)
NF/MHF	FF-SF	-0.096	-0.415 to 0.222	0.7580
	FF-LF	0.620	0.302 to 0.939	< 0.001***
	SF-LF	0.716	0.398 to 1.035	< 0.001***
UHF	FF-SF	0.009	-0.309 to 0.328	0.9975
	FF-LF	0.467	0.148 to 0.785	0.0017**
	SF-LF	0.458	0.139 to 0.776	0.0022**

ΔCSA = change in total cross-sectional area (mm²); ΔHU = change in mean z-standardized Hounsfield Unit values; CI = confidence interval; p-values adjusted using Tukey’s method for multiple comparisons. Statistically significant results are marked with * (*p* < 0.05), ** (*p* < 0.01), *** (*p* < 0.001)

#### Determinants of muscle change during radiotherapy

**Fig. 4:**
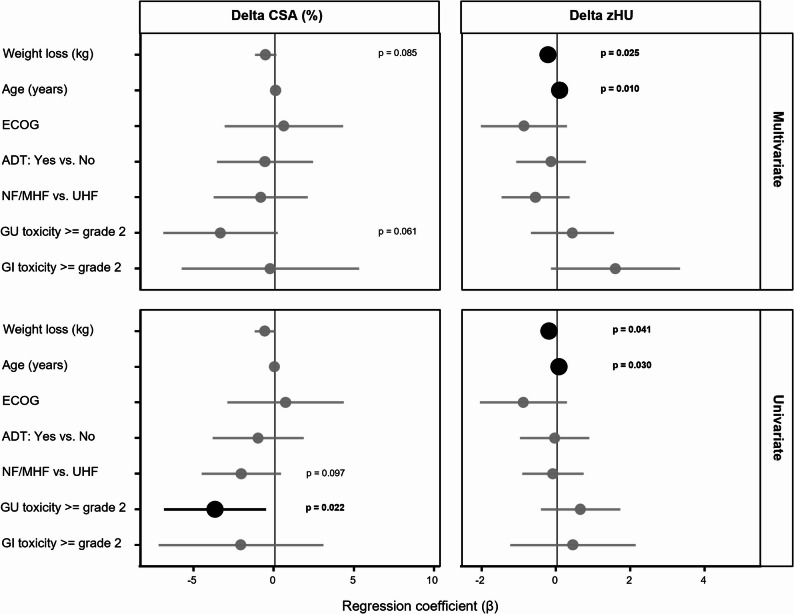
Forest plots of regression coefficients (β) with 95% confidence intervals (CI) for potential predictors of intratreatment muscle changes, expressed as ΔCSA (%) and ΔzHU between first and last treatment fraction. Separate panels depict univariate and multivariate linear regression models. A vertical reference line at β = 0 indicates no association. Negative coefficients indicate lower Δ values, corresponding to a greater decline from baseline, whereas positive coefficients indicate higher Δ values, corresponding to a smaller decline or relative preservation. Statistically significant predictors (*p* < 0.05) are labeled. Predictors included weight loss during radiotherapy, patient age, ECOG performance status, androgen deprivation therapy (ADT), fractionation regimen (normofractionated&mild-/moderately hypofractionated [NF/MHF] vs. ultrahypofractionated [UHF]), and acute genitourinary (GU) or gastrointestinal (GI) toxicity ≥ grade 2. CSA = cross-sectional area; zHU = z-standardized Hounsfield Units; ADT = androgen deprivation therapy; GI = gastrointestinal; GU = genitourinary; ECOG = Eastern Cooperative Oncology Group performance status; β = regression coefficient; CI = confidence interval

To evaluate whether clinical or treatment-related factors influenced intratreatment muscle alterations, univariate and multivariate linear regression analyses were performed using ΔCSA (%) and ΔzHU between the first and last treatment fraction as dependent variables (Fig. 4).

In univariate analyses, greater weight loss during radiotherapy was associated with lower ΔzHU values, corresponding to a stronger decline in muscle density (β = −0.22, 95% CI − 0.42 to − 0.01, *p* = 0.041). In contrast, older age was associated with higher ΔzHU values, indicating a smaller decline in muscle density (β = 0.05, 95% CI 0.01 to 0.10, *p* = 0.030). No significant associations were observed for ADT use, ECOG performance status, or fractionation regimen (NF/MHF vs. UHF).

For ΔCSA, the occurrence of acute GU toxicity ≥ grade 2 was associated with lower ΔCSA values, corresponding to a greater decline in muscle area in univariate analysis (β = −3.73, 95% CI − 6.92 to − 0.55, *p* = 0.022). Weight loss showed a borderline association (β = −0.63, 95% CI − 1.26 to 0.01, *p* = 0.052). In addition, fractionation regimen demonstrated a borderline effect, with the NF/MHF cohort showing a stronger decline in ΔCSA compared to UHF (β = −2.10, 95% CI − 4.58 to 0.38, *p* = 0.097). No other variables, including age, ADT, and ECOG performance status, were significantly associated with ΔCSA.

In multivariate analysis, greater weight loss (β = −0.24, 95% CI − 0.45 to − 0.03, *p* = 0.025) remained independently associated with lower ΔzHU values, indicating a stronger decline in muscle density. Older age remained independently associated with higher ΔzHU values (β = 0.07, 95% CI 0.02 to 0.12, *p* = 0.010), corresponding to a smaller decline in muscle density. In contrast, neither ADT use nor fractionation regimen showed a significant association with ΔzHU.

No independent predictors of ΔCSA were identified in the multivariable model, although GU toxicity ≥ grade 2 showed a borderline association (β = −3.40, 95% CI − 6.96 to 0.17, *p* = 0.061).

#### Association of muscle loss with acute toxicity

Univariate and multivariate logistic regression analyses were performed to identify predictors of acute GU and GI toxicity occurring during the radiotherapy course. Both dichotomous (> 5%) and continuous (%) representations of CSA loss were evaluated in separate models. In the NF/MHF cohort, a decline in cross-sectional muscle area greater than 5% was significantly associated with acute GU toxicity in univariate analysis (odds ratio 4.90, 95% CI 1.17–22.7, *p* = 0.033). Continuous CSA loss showed a non-significant trend toward association with acute GU toxicity (OR 1.14 per % loss, 95% CI 0.99–1.35, *p* = 0.082). No association was observed for GI toxicity. In the UHF cohort, none of the tested variables reached statistical significance for GU toxicity, whereas GI toxicity could not be analyzed due to the absence of events. After adjustment for age, weight loss, ECOG performance status, and ADT, neither dichotomous CSA loss > 5% (OR 3.57, 95% CI 0.75–18.7, *p* = 0.115) nor continuous CSA loss (OR 1.12 per % loss, 95% CI 0.97–1.31, *p* = 0.146) remained statistically significant in the NF/MHF cohort, and no covariate emerged as an independent predictor of acute GU or GI toxicity. Fisher’s exact test confirmed an unadjusted association between CSA loss > 5% and GU toxicity in the NF/MHF group (*p* = 0.014), while no significant associations were observed for GI toxicity or in the UHF cohort.

**Fig. 5:**
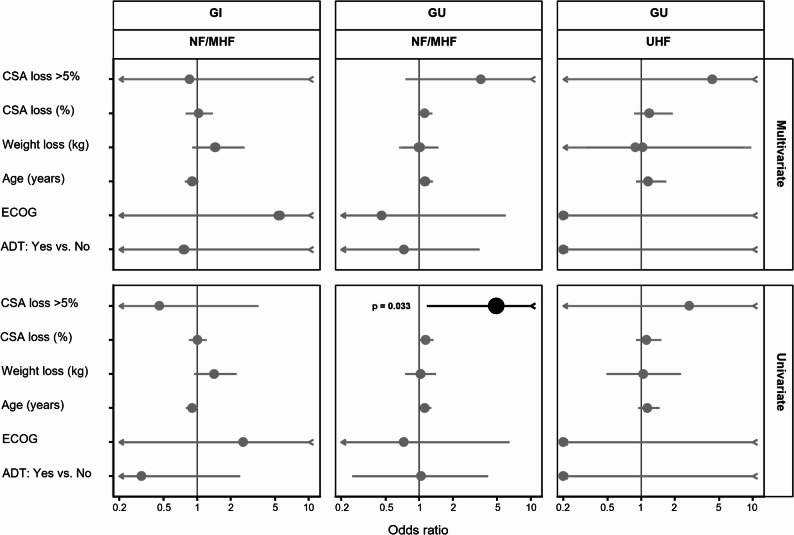
Forest plots of odds ratios (OR) with 95% confidence intervals (CI) for potential predictors of acute GU and GI toxicity (RTOG ≥ grade 2) in normofractionated&mild-/moderately hypofractionated (NF/MHF) and ultrahypofractionated (UHF) cohorts. Separate panels depict univariate and multivariate models. A vertical reference line at OR = 1 indicates no association. Significant predictors (*p* < 0.05) are labeled. Truncated confidence intervals extending beyond the plotting range (0.25-10) are marked with arrowheads. GI toxicity (UHF) not estimable due to absence of events. ADT effect in UHF not estimable because the confidence interval diverged (perfect separation). CSA loss was analyzed both as a binary variable (> 5%) and as a continuous percentage. CSA = cross-sectional area; ADT = androgen deprivation therapy; GI = gastrointestinal; GU = genitourinary; ECOG = Eastern Cooperative Oncology Group performance status; OR = odds ratio; CI = confidence interval

Figure 5 summarizes the corresponding odds ratios and 95% confidence intervals for all predictors across univariate and multivariate models.

## Discussion

This study is the first to validate skeletal muscle assessments from HyperSight™ CBCT in prostate cancer patients, a setting in which daily CBCT is routinely acquired for image guidance. We confirmed excellent agreement between planning CT and CBCT-derived CSA, supporting the feasibility of body composition analysis from HyperSight™ CBCT based image-guidance scans.

Consistent with Huang et al., who showed in head and neck cancer patients that CBCT enables reliable assessment of cervical muscle CSA, we observed similar feasibility with HyperSight™ imaging. Beyond their CSA-only approach, our use of z-standardized HU analysis further revealed a significant decline in muscle density for different dose concepts, an insight not identified before in prostate cancer patients [15].

The subsequent analysis of this study focused on longitudinal changes in muscle area and density and their clinical correlates during treatment. While a decline in muscle CSA was primarily observed in the NF/MHF cohort, z-standardized HU values decreased consistently in both cohorts, including UHF. Importantly, the ΔzHU scores did not differ significantly between the two groups, suggesting that HU-based muscle quality deterioration occurs in a similar magnitude across fractionation concepts and may represent a more uniform or earlier marker of treatment-related change than CSA. The magnitude of change was modest compared to reports from catabolic populations [31, 32]. Kiss et al. observed a pronounced early decline in skeletal muscle area (− 6.4 cm² at L3 within the first four weeks of concurrent chemoradiation), accompanied by a parallel reduction in muscle density (− 1.3 HU over the same period), both of which remained largely unchanged during subsequent follow-up reflecting the substantial systemic inflammatory and metabolic burden induced by combined chemotherapy and thoracic irradiation [32]. In contrast, our prostate cancer cohort underwent radiotherapy alone, typically associated with less systemic inflammation and metabolic stress, which may explain the smaller overall magnitude of decline. In the NF/MHF cohort, the longer treatment course may further contribute to this effect, as patients often experience a temporary reduction in daily physical activity during prolonged, appointment-centered treatment periods. Preliminary evidence suggests that physical exercise interventions, particularly resistance-based training, may help counteract sarcopenia by supporting muscle mass, strength, and physical function in cancer populations, including men with prostate cancer [33]. Accordingly, the potential benefits of proactive physical activity should be evaluated, particularly in these patients. Moreover, our measurements were derived from CBCT rather than diagnostic CT. HyperSight™ CBCT provides high soft-tissue contrast and benefits from advanced reconstruction techniques, and because it is acquired routinely for daily IGRT, it represents a practical tool for longitudinal body composition monitoring without additional scans or radiation exposure [34]. By applying z-score normalization relative to subcutaneous fat, we compensated for scanner-dependent variability and were able to detect subtle, treatment-related reductions in muscle density.

In response to the potential confounding effects of treatment concept and hormonal therapy, we first examined if ADT and radiotherapy fractionation regimen were associated with intratreatment muscle decline using linear regression models. In these analyses, neither ADT use nor fractionation regimen emerged as independent significant predictors of changes in muscle area (ΔCSA) or muscle density (ΔzHU). ADT was considered a potential cofactor in earlier studies [13, 14], but not in the results presented here. This may be due to the evaluation period during radiotherapy. Further, contrary to the increased decline of CSA in the NF/MHF cohort in the linear mixed-effects model assessment, the fractionation regimen showed only a borderline association with ΔCSA in the univariate regression analysis. However, this also indicated a greater decline in the NF/MHF cohort. The differing results compared to the longitudinal analysis may reflect methodological differences, as the mixed-effects model accounted for repeated measurements over time, whereas the regression analysis was based on a single summary measure between first and last fraction. Therefore, the association between sarcopenia and the fractionation regimen should be interpreted with caution. Further validation in larger cohorts is required.

Instead, patient-related factors appeared to play a more relevant role. Greater weight loss during radiotherapy was independently associated with lower ΔzHU values, corresponding to a stronger decline in muscle density. This finding is biologically plausible, as weight loss during treatment may reflect reduced caloric intake, systemic catabolic processes, or decreased physical activity, all of which can contribute to deterioration in muscle quality [2, 31, 32]. In contrast, older age was associated with higher ΔzHU values, indicating a smaller decline in muscle density. Future investigations should assess the impact of associated cofactors and characteristics of different patient groups, such as geriatric patients compared to younger patients, on the baseline status and longitudinal course of muscle composition. However, the magnitude of this effect was modest, and its clinical relevance remains uncertain. Notably, no independent predictors of ΔCSA were identified, suggesting that muscle density alterations may represent a more sensitive marker of early treatment-related changes than CSA alone.

We next evaluated the association between intratreatment muscle loss and acute treatment-related toxicity using univariate and multivariable logistic regression models. In the normofractionated&mild-/moderately hypofractionated cohort, binary CSA loss > 5% was associated with acute genitourinary toxicity in univariate analysis, whereas continuous CSA loss showed only a non-significant trend. However, these associations were not confirmed after multivariable adjustment, and no significant predictors of gastrointestinal toxicity were identified. In the ultrahypofractionated cohort, no associations between muscle loss and acute toxicity were observed.

Taken together, these analyses suggest that intratreatment muscle alterations cannot be fully explained by treatment-related factors alone and may also reflect interindividual differences in physiological response during radiotherapy. While prior studies across various tumor entities have reported associations between sarcopenia and increased treatment-related toxicity [35–38], the transferability of these findings to prostate cancer populations and the different monitoring periods remains uncertain. In this context, our results indicate that CBCT-derived changes in muscle density may capture early treatment-related vulnerability more sensitively than CSA alone, whereas associations between muscle loss and acute toxicity remain exploratory and hypothesis-generating, thus requiring confirmation in larger cohorts and longer observation periods.

It is important to note that the absence of any detectable association in the UHF cohort may potentially reflect intrinsic characteristics of ultrahypofractionated radiotherapy rather than a biological difference. Acute GU symptoms in UHF often peak only after treatment completion, meaning that the clinically most relevant toxicity window occurred at a time when no further CBCT scans were available. In the PACE-B and PACE-A trials, most grade ≥ 2 GU toxicities after ultrahypofractionated prostate radiotherapy were observed around day 7–10 post-treatment, rather than during the treatment week itself [39, 40]. This delay indicates that our imaging schedule may not have captured the period in which potential early muscle decline and symptom onset could have coincided.

In prostate cancer specifically, the evidence has been less uniform. In contrast to our findings, the recent analysis by Vickers et al., which relied on a single baseline planning CT, did not reveal any link between sarcopenia and radiotherapy-related toxicities [9]. This discrepancy underscores that static, CSA-based assessments may be insufficient in comparatively healthier patient cohorts, where dynamic, treatment-induced changes provide greater discriminatory value. Our results suggest that longitudinal muscle monitoring during radiotherapy may capture these subtle but clinically relevant alterations. With HyperSight™ CBCT enabling longitudinal zHU measurement, changes in muscle density may represent an even more sensitive marker of early treatment-related alterations than CSA alone. Leveraging daily CBCT imaging to monitor both CSA and HU decline could therefore support earlier identification of patients at risk for toxicity and facilitate timely nutritional or rehabilitative interventions.

Several limitations should be acknowledged. The main limitation arises from the different length of treatment regimens. UHF schedules compress both muscle assessment and toxicity recording into a very short time window, so that treatment-related muscle changes and the peak of acute toxicity may not occur concurrently. This temporal mismatch inherently reduces the comparability with NF/MHF regimens and may obscure associations that would otherwise become detectable over a longer treatment course.

Further limitations include the single-center design, the small sample size and the relatively homogeneous patient cohort, which may limit generalizability. ADT exposure was not uniform, and potential interactions with muscle change could not be fully assessed. Differences in slice thickness between planning CT (3 mm) and CBCT (2 mm), as well as differing scan parameters and reconstruction algorithms between PCT and CBCT, may have introduced minor variability in CSA measurements and contributed to the small systematic bias observed in the CT-CBCT comparison. The use of subcutaneous fat for HU normalization assumes relative stability during radiotherapy; however, fat may also change due to radiotherapy itself, weight loss or fluid shifts, potentially limiting its reliability as a reference. However, subcutaneous fat tissue, which is far from the high-dose region, may at least minimise this confounding factor as much as possible. In addition, the L3 reference level was not included in the CBCT field of view, precluding direct validation against the gold standard of body muscle composition; although a significant correlation with L4 muscle area was observed, the presented analysis should still be interpreted as a surrogate rather than a replacement for L3-based assessment. Moreover, further studies are required to investigate the reproducibility of segmentation for different muscles, involving different observers and various segmentation approaches. Finally, due to the small proportion of toxicity events in this cohort, a larger population with a longer follow-up period should be examined to more comprehensively evaluate potential associations. Nonetheless, the consistent direction of intra-treatment CBCT-derived muscle changes supports the robustness of the longitudinal findings.

## Conclusion

In conclusion, this study demonstrates the feasibility of skeletal muscle assessment using HyperSight™ CBCT in prostate cancer patients during radiotherapy. By jointly evaluating muscle area and density, we captured complementary aspects of treatment-related body composition change. In the NF/MHF cohort, declines in both CSA and HU were detectable, and CSA loss showed an exploratory association with acute GU toxicity in univariate analysis, but this was not confirmed after adjustment. Early reductions in z-standardized HU, observed across both fractionation concepts, may represent a sensitive marker of emerging muscle quality deterioration.

These findings highlight the potential of routine HyperSight™ CBCT for longitudinal muscle monitoring, and may support the further development of automated, artificial intelligence (AI)-driven pipelines for muscle segmentation and HU quantification, as is already possible for pelvic risk organs during adaptive radiotherapy [41]. Future work should also evaluate advanced quantitative imaging markers, including radiomic features [42], and explore composite parameters that integrate CSA and HU. Larger, multi-institutional studies including comparisons with established imaging modalities such as diagnostic CT or MRI, as well as extending analyses into post-treatment recovery, are warranted to clarify the clinical role of CBCT-derived muscle metrics.

## Data Availability

The data supporting the conclusion of this article may be made available, subject to ethical and data protection considerations, upon reasonable request on an individual basis. Please contact Constantin Dreher, MD (E-mail: constantin.dreher@medma.uni-heidelberg.de) to request the data.

## Abbreviations

ADT: Androgen deprivation therapy
BPL: Brilliance BigBore (Philips) reconstruction algorithm
CBCT: Cone–beam computed tomography
CCC: Concordance correlation coefficient
CI: Confidence interval
CSA: Cross–sectional area
CT: Computed tomography
CTDIvol: Volume CT dose index
DICOM: Digital Imaging and Communications in Medicine
DXA: Dual–energy X–ray absorptiometry
ECOG: Eastern Cooperative Oncology Group performance status
FBCT: Fan–beam computed tomography
FF: First fraction
GI: Gastrointestinal
GU: Genitourinary
HU: Hounsfield Units
IGRT: Image–guided radiotherapy
iCBCT: Iterative CBCT reconstruction
LF: Last fraction
MHF: Moderately hypofractionated (radiotherapy)
MRI: Magnetic resonance imaging
NF: Normofractionated (radiotherapy)
OR: Odds ratio
PCT: Planning computed tomography
ROI: Region of interest
RT: Radiotherapy
RTOG: Radiation Therapy Oncology Group
SBRT: Stereotactic body radiotherapy
SD: Standard deviation
SF: Second fraction
UHF: Ultrahypofractionated (radiotherapy)
zHU: z–standardized Hounsfield Unit
